# (2-{[1,1-Bis(hy­droxy­meth­yl)-2-oxidoeth­yl]imino­meth­yl}-4-chloro­phenolato-κ^3^ *O*,*N*,*O*′)dibutyl­tin(IV)

**DOI:** 10.1107/S1600536810021872

**Published:** 2010-06-16

**Authors:** See Mun Lee, Hapipah Mohd Ali, Kong Mun Lo

**Affiliations:** a Department of Chemistry, University of Malaya, 50603 Kuala Lumpur, Malaysia

## Abstract

In the title compound, [Sn(C_4_H_9_)_2_(C_11_H_12_BrNO_4_)], the Schiff base ligand chelates to the Sn^IV^ atom through the two deprotonated hy­droxy groups, as well as through the N atom, to confer an overall *cis*-C_2_SnNO_2_ trigonal-bipyramidal geometry at the Sn^IV^ atom [C—Sn—C = 129.92 (9)°]. The remaining methyl­enehy­droxy groups engage in O—H⋯O hydrogen bonding with the O atoms of adjacent mol­ecules, leading to infinite supra­molecular chains propagating in [001].

## Related literature

For related structures, see Reisi *et al.* (2010 ▶); Ng (2008 ▶).
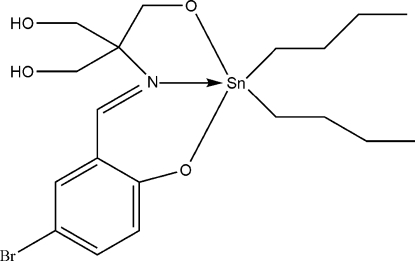


## Experimental

### 

#### Crystal data

[Sn(C_4_H_9_)_2_(C_11_H_12_BrNO_4_)]
*M* *_r_* = 535.04
Monoclinic, 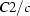

*a* = 18.8326 (9) Å
*b* = 13.3811 (7) Å
*c* = 16.5768 (8) Å
β = 91.385 (3)°
*V* = 4176.1 (4) Å^3^
*Z* = 8
Mo *K*α radiation
μ = 3.16 mm^−1^
*T* = 100 K
0.40 × 0.10 × 0.08 mm

#### Data collection

Bruker APEXII CCD area-detector diffractometer
Absorption correction: multi-scan (*SADABS*; Bruker, 2009 ▶) *T*_min_ = 0.365, *T*_max_ = 0.786
19535 measured reflections
4785 independent reflections
4229 reflections with *I* > 2σ(*I*)
*R*_int_ = 0.032

#### Refinement

*R*[*F* ^2^ > 2σ(*F* ^2^)] = 0.022
*wR*(*F* ^2^) = 0.052
*S* = 1.02
4785 reflections
239 parameters
2 restraints
H-atom parameters constrained
Δρ_max_ = 0.65 e Å^−3^
Δρ_min_ = −0.38 e Å^−3^

### 

Data collection: *APEX2* (Bruker, 2009 ▶); cell refinement: *SAINT* (Bruker, 2009 ▶); data reduction: *SAINT*; program(s) used to solve structure: *SHELXS97* (Sheldrick, 2008 ▶); program(s) used to refine structure: *SHELXL97* (Sheldrick, 2008 ▶); molecular graphics: *X-SEED* (Barbour, 2001 ▶); software used to prepare material for publication: *pubCIF* (Westrip, 2010 ▶).

## Supplementary Material

Crystal structure: contains datablocks I, global. DOI: 10.1107/S1600536810021872/xu2772sup1.cif

Structure factors: contains datablocks I. DOI: 10.1107/S1600536810021872/xu2772Isup2.hkl

Additional supplementary materials:  crystallographic information; 3D view; checkCIF report

## Figures and Tables

**Table 1:** Selected bond lengths (Å)

Sn1—N1	2.2108 (17)
Sn1—O1	2.1203 (15)
Sn1—O2	2.1049 (14)
Sn1—C12	2.139 (2)
Sn1—C16	2.129 (2)

**Table 2:** Hydrogen-bond geometry (Å, °)

*D*—H⋯*A*	*D*—H	H⋯*A*	*D*⋯*A*	*D*—H⋯*A*
O3—H3⋯O2^i^	0.84	1.77	2.608 (2)	174
O4—H4⋯O3^ii^	0.84	1.93	2.733 (2)	160

Symmetry codes: (i) 
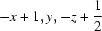
; (ii) 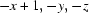.

**Fig. 1.**
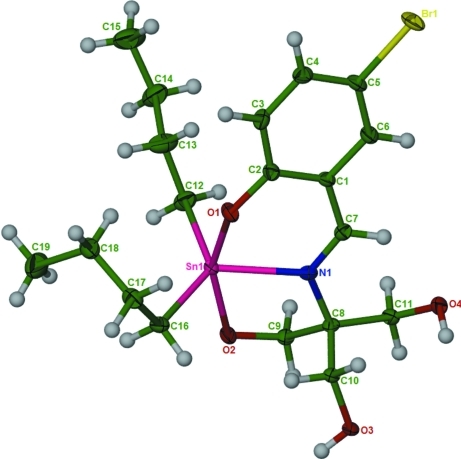
The molecular structure of (2-{[1,1-bis(hydroxymethyl)-2-oxidoethyl]iminomethyl}-4-chlorophenolato-κ3N,O,O')dibutyltin(IV) showing 70% probability displacement ellipsoids and the atom numbering. Hydrogen atoms are drawn as spheres of arbitrary radius.

**Fig. 2.**
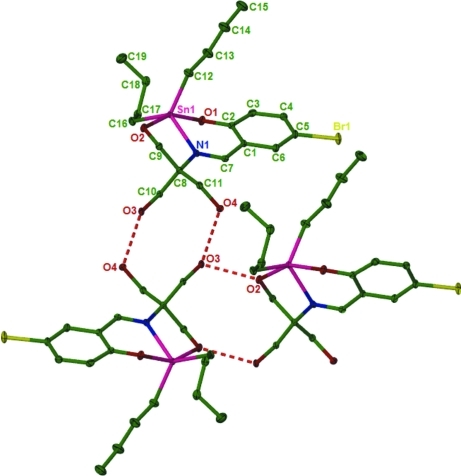
Crystal packing of the unit cell showing the hydrogen bonding interactions in the molecule.

## supplementary crystallographic information

### Comment

The Schiff base derived from the condensation of 5-bromosalicylaldehyde and tris(hydroxymethyl)methylamine is deprotonated with respect to the phenoxy hydrogen atom and one of the methylenehydroxyl hydrogen atom. The ligand coordinates to the dibutyltin fragment through this doubly deprotonated oxygen atoms and the imine nitrogen (Fig. 1).

The tin atom is in a *cis*-trigonal bipyramidal geometry with a C—Sn—C angle of 129.92 (9)°. The two deprotonated oxygen atoms occupied the axial sites with a O—Sn—O angle of 155.60 (6)°, indicating a distortion in the trigonal bipyramidal geometry at the Sn atom. Adjacent molecules are linked by hydrogen bonds to form an infinite polymeric chain (Fig. 2).

### Experimental

The Schiff base, 4-bromo-2-tris[(hydroxymethyl)methylimino]phenol was prepared from tris(hydroxymethyl)aminomethane and 5-bromosalicylaldehyde in absolute ethanol. The compound (0.30 g, 0.1 mmol) and dibutyltin oxide (0.25 g, 1.0 mmol) were heated in 50 ml of toluene in a Dean-Stark apparatus for 8 h. The solution was left for crystallizaton for a week during which yellow crystals were obtained.

### Refinement

Hydrogen atoms were placed at calculated positions (C–H 0.95 to 0.98 Å) and were treated as riding on their parent carbon atoms, with *U*_iso_(H) set to 1.2–1.5 times *U*_eq_(C). The hydroxy-H was refined with a restraint of 0.84 ± 0.01 Å, U_iso_(H) = 1.5U_eq_(O).

### Crystal data

**Table:**

[Sn(C_4_H_9_)_2_(C_11_H_12_BrNO_4_)]	*F*(000) = 2144
*M**_r_* = 535.04	*D*_x_ = 1.702 Mg m^−^^3^
Monoclinic, *C*2/*c*	Mo *K*α radiation, λ = 0.71073 Å
Hall symbol: -C 2yc	Cell parameters from 7855 reflections
*a* = 18.8326 (9) Å	θ = 2.2–30.4°
*b* = 13.3811 (7) Å	µ = 3.16 mm^−^^1^
*c* = 16.5768 (8) Å	*T* = 100 K
β = 91.385 (3)°	Needle, yellow
*V* = 4176.1 (4) Å^3^	0.40 × 0.10 × 0.08 mm
*Z* = 8	

### Data collection

**Table:**

Bruker APEXII CCD area-detector diffractometer	4785 independent reflections
Radiation source: fine-focus sealed tube	4229 reflections with *I* > 2σ(*I*)
graphite	*R*_int_ = 0.032
ω scans	θ_max_ = 27.5°, θ_min_ = 1.9°
Absorption correction: multi-scan (*SADABS*; Bruker, 2009)	*h* = −24→24
*T*_min_ = 0.365, *T*_max_ = 0.786	*k* = −17→17
19535 measured reflections	*l* = −21→21

### Refinement

**Table:**

Refinement on *F*^2^	Primary atom site location: structure-invariant direct methods
Least-squares matrix: full	Secondary atom site location: difference Fourier map
*R*[*F*^2^ > 2σ(*F*^2^)] = 0.022	Hydrogen site location: inferred from neighbouring sites
*wR*(*F*^2^) = 0.052	H-atom parameters constrained
*S* = 1.02	*w* = 1/[σ^2^(*F*_o_^2^) + (0.0231*P*)^2^ + 4.784*P*] where *P* = (*F*_o_^2^ + 2*F*_c_^2^)/3
4785 reflections	(Δ/σ)_max_ = 0.001
239 parameters	Δρ_max_ = 0.65 e Å^−^^3^
2 restraints	Δρ_min_ = −0.37 e Å^−^^3^

### Special details

**Table:**

Geometry. All e.s.d.'s (except the e.s.d. in the dihedral angle between two l.s. planes) are estimated using the full covariance matrix. The cell e.s.d.'s are taken into account individually in the estimation of e.s.d.'s in distances, angles and torsion angles; correlations between e.s.d.'s in cell parameters are only used when they are defined by crystal symmetry. An approximate (isotropic) treatment of cell e.s.d.'s is used for estimating e.s.d.'s involving l.s. planes.
Refinement. Refinement of *F*^2^ against ALL reflections. The weighted *R*-factor *wR* and goodness of fit *S* are based on *F*^2^, conventional *R*-factors *R* are based on *F*, with *F* set to zero for negative *F*^2^. The threshold expression of *F*^2^ > σ(*F*^2^) is used only for calculating *R*-factors(gt) *etc*. and is not relevant to the choice of reflections for refinement. *R*-factors based on *F*^2^ are statistically about twice as large as those based on *F*, and *R*- factors based on ALL data will be even larger.

### Fractional atomic coordinates and isotropic or equivalent isotropic displacement parameters (Å^2^)

**Table:**

	*x*	*y*	*z*	*U*_iso_*/*U*_eq_	
Sn1	0.325854 (7)	0.128538 (10)	0.254101 (8)	0.01133 (5)	
Br1	0.047899 (12)	0.180095 (17)	−0.072373 (14)	0.02090 (6)	
N1	0.33348 (9)	0.06301 (13)	0.13219 (10)	0.0117 (3)	
O1	0.27217 (8)	0.24249 (11)	0.18796 (9)	0.0154 (3)	
O2	0.39697 (8)	0.00855 (11)	0.27086 (8)	0.0141 (3)	
O3	0.51910 (7)	−0.01628 (11)	0.10327 (9)	0.0139 (3)	
H3	0.5442	−0.0111	0.1456	0.021*	
O4	0.37130 (8)	−0.06319 (12)	−0.01932 (8)	0.0151 (3)	
H4	0.4107	−0.0416	−0.0344	0.023*	
C1	0.22983 (11)	0.14839 (15)	0.07343 (12)	0.0119 (4)	
C2	0.22471 (11)	0.22806 (16)	0.12949 (12)	0.0132 (4)	
C3	0.16695 (12)	0.29422 (16)	0.12036 (12)	0.0153 (4)	
H3A	0.1638	0.3502	0.1554	0.018*	
C4	0.11478 (11)	0.27967 (17)	0.06175 (13)	0.0155 (4)	
H4A	0.0751	0.3234	0.0581	0.019*	
C5	0.12066 (11)	0.19991 (16)	0.00753 (12)	0.0148 (4)	
C6	0.17791 (11)	0.13705 (16)	0.01159 (13)	0.0144 (4)	
H6	0.1826	0.0856	−0.0274	0.017*	
C7	0.28804 (11)	0.07801 (16)	0.07408 (12)	0.0125 (4)	
H7	0.2933	0.0388	0.0268	0.015*	
C8	0.39114 (11)	−0.01213 (15)	0.12512 (12)	0.0116 (4)	
C9	0.39651 (11)	−0.06281 (16)	0.20865 (12)	0.0130 (4)	
H9A	0.3557	−0.1085	0.2150	0.016*	
H9B	0.4406	−0.1030	0.2124	0.016*	
C10	0.45890 (10)	0.04737 (15)	0.10849 (12)	0.0117 (4)	
H10A	0.4525	0.0847	0.0573	0.014*	
H10B	0.4672	0.0966	0.1523	0.014*	
C11	0.37745 (11)	−0.09448 (16)	0.06228 (12)	0.0131 (4)	
H11A	0.4167	−0.1436	0.0668	0.016*	
H11B	0.3331	−0.1296	0.0762	0.016*	
C12	0.23527 (12)	0.06420 (17)	0.30964 (14)	0.0191 (5)	
H12A	0.2208	0.0044	0.2781	0.023*	
H12B	0.2500	0.0410	0.3642	0.023*	
C13	0.17028 (12)	0.13118 (19)	0.31788 (16)	0.0259 (5)	
H13A	0.1816	0.1846	0.3574	0.031*	
H13B	0.1597	0.1635	0.2652	0.031*	
C14	0.10488 (13)	0.07612 (19)	0.34454 (17)	0.0272 (5)	
H14A	0.1168	0.0397	0.3951	0.033*	
H14B	0.0918	0.0258	0.3030	0.033*	
C15	0.04070 (14)	0.1421 (2)	0.35880 (18)	0.0350 (7)	
H15A	0.0495	0.1833	0.4069	0.052*	
H15B	−0.0012	0.1001	0.3669	0.052*	
H15C	0.0323	0.1854	0.3119	0.052*	
C16	0.40219 (11)	0.23185 (17)	0.30125 (13)	0.0164 (4)	
H16A	0.4161	0.2102	0.3565	0.020*	
H16B	0.4451	0.2276	0.2680	0.020*	
C17	0.37945 (12)	0.34137 (17)	0.30495 (14)	0.0180 (5)	
H17A	0.4205	0.3818	0.3243	0.022*	
H17B	0.3667	0.3641	0.2496	0.022*	
C18	0.31701 (12)	0.36167 (17)	0.35918 (14)	0.0200 (5)	
H18A	0.3050	0.4336	0.3560	0.024*	
H18B	0.2753	0.3237	0.3385	0.024*	
C19	0.33061 (15)	0.3340 (2)	0.44694 (15)	0.0344 (7)	
H19A	0.3368	0.2615	0.4515	0.052*	
H19B	0.2901	0.3549	0.4789	0.052*	
H19C	0.3737	0.3677	0.4671	0.052*	

### Atomic displacement parameters (Å^2^)

**Table:**

	*U*^11^	*U*^22^	*U*^33^	*U*^12^	*U*^13^	*U*^23^
Sn1	0.00935 (7)	0.01444 (8)	0.01012 (7)	0.00111 (5)	−0.00151 (5)	−0.00132 (5)
Br1	0.01670 (11)	0.02112 (12)	0.02432 (12)	0.00431 (9)	−0.01116 (9)	−0.00292 (9)
N1	0.0092 (8)	0.0119 (8)	0.0140 (8)	0.0008 (7)	0.0000 (6)	0.0013 (7)
O1	0.0171 (8)	0.0148 (7)	0.0139 (7)	0.0022 (6)	−0.0048 (6)	−0.0024 (6)
O2	0.0146 (7)	0.0160 (7)	0.0116 (7)	0.0034 (6)	−0.0035 (6)	−0.0013 (6)
O3	0.0092 (7)	0.0196 (8)	0.0127 (7)	0.0046 (6)	−0.0028 (6)	−0.0026 (6)
O4	0.0133 (7)	0.0214 (8)	0.0107 (7)	−0.0014 (6)	0.0005 (6)	−0.0005 (6)
C1	0.0097 (9)	0.0143 (10)	0.0117 (10)	0.0006 (8)	−0.0013 (8)	0.0013 (8)
C2	0.0128 (10)	0.0164 (10)	0.0105 (9)	−0.0011 (8)	0.0001 (8)	0.0010 (8)
C3	0.0185 (11)	0.0149 (10)	0.0126 (10)	0.0024 (9)	0.0015 (8)	−0.0015 (8)
C4	0.0131 (10)	0.0190 (11)	0.0144 (10)	0.0034 (9)	0.0003 (8)	0.0031 (8)
C5	0.0117 (10)	0.0188 (11)	0.0138 (10)	−0.0011 (9)	−0.0042 (8)	0.0028 (8)
C6	0.0140 (10)	0.0154 (10)	0.0136 (10)	0.0012 (8)	−0.0023 (8)	−0.0004 (8)
C7	0.0119 (10)	0.0128 (10)	0.0129 (10)	−0.0002 (8)	0.0018 (8)	0.0001 (8)
C8	0.0096 (9)	0.0127 (10)	0.0124 (10)	0.0032 (8)	−0.0003 (7)	0.0003 (8)
C9	0.0120 (10)	0.0141 (10)	0.0128 (10)	0.0027 (8)	−0.0009 (8)	0.0001 (8)
C10	0.0095 (9)	0.0140 (10)	0.0114 (10)	0.0009 (8)	−0.0010 (7)	−0.0002 (8)
C11	0.0110 (9)	0.0144 (10)	0.0138 (10)	−0.0006 (8)	−0.0008 (8)	−0.0009 (8)
C12	0.0155 (11)	0.0192 (11)	0.0227 (12)	−0.0013 (9)	0.0043 (9)	−0.0017 (9)
C13	0.0166 (12)	0.0316 (14)	0.0298 (13)	0.0049 (10)	0.0043 (10)	0.0099 (11)
C14	0.0196 (12)	0.0264 (13)	0.0359 (14)	−0.0035 (11)	0.0077 (10)	−0.0110 (11)
C15	0.0179 (13)	0.0498 (18)	0.0376 (16)	0.0035 (12)	0.0062 (11)	0.0078 (13)
C16	0.0115 (10)	0.0193 (11)	0.0183 (11)	0.0024 (9)	−0.0016 (8)	−0.0034 (9)
C17	0.0177 (11)	0.0174 (11)	0.0188 (11)	−0.0003 (9)	−0.0035 (9)	−0.0014 (9)
C18	0.0201 (11)	0.0197 (11)	0.0199 (11)	0.0051 (9)	−0.0042 (9)	−0.0049 (9)
C19	0.0313 (14)	0.0536 (18)	0.0184 (12)	0.0169 (14)	0.0004 (11)	0.0000 (12)

### Geometric parameters (Å, °)

**Table:**

Sn1—N1	2.2108 (17)	C9—H9B	0.9900
Sn1—O1	2.1203 (15)	C10—H10A	0.9900
Sn1—O2	2.1049 (14)	C10—H10B	0.9900
Sn1—C12	2.139 (2)	C11—H11A	0.9900
Sn1—C16	2.129 (2)	C11—H11B	0.9900
Br1—C5	1.901 (2)	C12—C13	1.526 (3)
N1—C7	1.289 (3)	C12—H12A	0.9900
N1—C8	1.487 (3)	C12—H12B	0.9900
O1—C2	1.317 (2)	C13—C14	1.510 (3)
O2—C9	1.405 (2)	C13—H13A	0.9900
O3—C10	1.422 (2)	C13—H13B	0.9900
O3—H3	0.8400	C14—C15	1.520 (3)
O4—C11	1.418 (2)	C14—H14A	0.9900
O4—H4	0.8400	C14—H14B	0.9900
C1—C6	1.408 (3)	C15—H15A	0.9800
C1—C2	1.419 (3)	C15—H15B	0.9800
C1—C7	1.445 (3)	C15—H15C	0.9800
C2—C3	1.408 (3)	C16—C17	1.528 (3)
C3—C4	1.379 (3)	C16—H16A	0.9900
C3—H3A	0.9500	C16—H16B	0.9900
C4—C5	1.401 (3)	C17—C18	1.522 (3)
C4—H4A	0.9500	C17—H17A	0.9900
C5—C6	1.368 (3)	C17—H17B	0.9900
C6—H6	0.9500	C18—C19	1.517 (3)
C7—H7	0.9500	C18—H18A	0.9900
C8—C11	1.534 (3)	C18—H18B	0.9900
C8—C10	1.535 (3)	C19—H19A	0.9800
C8—C9	1.543 (3)	C19—H19B	0.9800
C9—H9A	0.9900	C19—H19C	0.9800
			
O2—Sn1—O1	155.60 (6)	H10A—C10—H10B	108.0
O2—Sn1—C16	91.43 (7)	O4—C11—C8	116.38 (17)
O1—Sn1—C16	91.84 (7)	O4—C11—H11A	108.2
O2—Sn1—C12	98.49 (7)	C8—C11—H11A	108.2
O1—Sn1—C12	97.86 (8)	O4—C11—H11B	108.2
C16—Sn1—C12	129.92 (9)	C8—C11—H11B	108.2
O2—Sn1—N1	76.29 (6)	H11A—C11—H11B	107.3
O1—Sn1—N1	81.56 (6)	C13—C12—Sn1	116.87 (16)
C16—Sn1—N1	122.33 (7)	C13—C12—H12A	108.1
C12—Sn1—N1	107.69 (8)	Sn1—C12—H12A	108.1
C7—N1—C8	121.31 (18)	C13—C12—H12B	108.1
C7—N1—Sn1	124.39 (14)	Sn1—C12—H12B	108.1
C8—N1—Sn1	113.83 (12)	H12A—C12—H12B	107.3
C2—O1—Sn1	125.58 (13)	C14—C13—C12	113.7 (2)
C9—O2—Sn1	115.39 (12)	C14—C13—H13A	108.8
C10—O3—H3	109.5	C12—C13—H13A	108.8
C11—O4—H4	109.5	C14—C13—H13B	108.8
C6—C1—C2	120.06 (19)	C12—C13—H13B	108.8
C6—C1—C7	116.66 (19)	H13A—C13—H13B	107.7
C2—C1—C7	123.25 (19)	C13—C14—C15	114.8 (2)
O1—C2—C3	119.74 (19)	C13—C14—H14A	108.6
O1—C2—C1	122.45 (19)	C15—C14—H14A	108.6
C3—C2—C1	117.81 (19)	C13—C14—H14B	108.6
C4—C3—C2	121.5 (2)	C15—C14—H14B	108.6
C4—C3—H3A	119.2	H14A—C14—H14B	107.5
C2—C3—H3A	119.2	C14—C15—H15A	109.5
C3—C4—C5	119.6 (2)	C14—C15—H15B	109.5
C3—C4—H4A	120.2	H15A—C15—H15B	109.5
C5—C4—H4A	120.2	C14—C15—H15C	109.5
C6—C5—C4	120.8 (2)	H15A—C15—H15C	109.5
C6—C5—Br1	120.16 (16)	H15B—C15—H15C	109.5
C4—C5—Br1	119.09 (16)	C17—C16—Sn1	116.73 (14)
C5—C6—C1	120.2 (2)	C17—C16—H16A	108.1
C5—C6—H6	119.9	Sn1—C16—H16A	108.1
C1—C6—H6	119.9	C17—C16—H16B	108.1
N1—C7—C1	126.66 (19)	Sn1—C16—H16B	108.1
N1—C7—H7	116.7	H16A—C16—H16B	107.3
C1—C7—H7	116.7	C18—C17—C16	114.59 (19)
N1—C8—C11	115.35 (16)	C18—C17—H17A	108.6
N1—C8—C10	105.98 (16)	C16—C17—H17A	108.6
C11—C8—C10	112.19 (16)	C18—C17—H17B	108.6
N1—C8—C9	104.99 (15)	C16—C17—H17B	108.6
C11—C8—C9	107.46 (17)	H17A—C17—H17B	107.6
C10—C8—C9	110.64 (16)	C19—C18—C17	114.1 (2)
O2—C9—C8	111.04 (17)	C19—C18—H18A	108.7
O2—C9—H9A	109.4	C17—C18—H18A	108.7
C8—C9—H9A	109.4	C19—C18—H18B	108.7
O2—C9—H9B	109.4	C17—C18—H18B	108.7
C8—C9—H9B	109.4	H18A—C18—H18B	107.6
H9A—C9—H9B	108.0	C18—C19—H19A	109.5
O3—C10—C8	111.57 (16)	C18—C19—H19B	109.5
O3—C10—H10A	109.3	H19A—C19—H19B	109.5
C8—C10—H10A	109.3	C18—C19—H19C	109.5
O3—C10—H10B	109.3	H19A—C19—H19C	109.5
C8—C10—H10B	109.3	H19B—C19—H19C	109.5
			
O2—Sn1—N1—C7	161.51 (18)	Sn1—N1—C7—C1	8.4 (3)
O1—Sn1—N1—C7	−28.79 (17)	C6—C1—C7—N1	−166.4 (2)
C16—Sn1—N1—C7	−115.61 (17)	C2—C1—C7—N1	15.8 (3)
C12—Sn1—N1—C7	66.82 (18)	C7—N1—C8—C11	−22.0 (3)
O2—Sn1—N1—C8	−10.66 (12)	Sn1—N1—C8—C11	150.45 (14)
O1—Sn1—N1—C8	159.04 (14)	C7—N1—C8—C10	102.8 (2)
C16—Sn1—N1—C8	72.22 (15)	Sn1—N1—C8—C10	−84.77 (15)
C12—Sn1—N1—C8	−105.35 (14)	C7—N1—C8—C9	−140.06 (19)
O2—Sn1—O1—C2	67.9 (2)	Sn1—N1—C8—C9	32.38 (18)
C16—Sn1—O1—C2	165.50 (16)	Sn1—O2—C9—C8	41.10 (19)
C12—Sn1—O1—C2	−63.76 (17)	N1—C8—C9—O2	−46.7 (2)
N1—Sn1—O1—C2	43.07 (16)	C11—C8—C9—O2	−169.96 (16)
O1—Sn1—O2—C9	−42.4 (2)	C10—C8—C9—O2	67.3 (2)
C16—Sn1—O2—C9	−140.00 (14)	N1—C8—C10—O3	177.90 (15)
C12—Sn1—O2—C9	89.25 (14)	C11—C8—C10—O3	−55.4 (2)
N1—Sn1—O2—C9	−17.01 (13)	C9—C8—C10—O3	64.6 (2)
Sn1—O1—C2—C3	144.75 (16)	N1—C8—C11—O4	63.8 (2)
Sn1—O1—C2—C1	−36.3 (3)	C10—C8—C11—O4	−57.7 (2)
C6—C1—C2—O1	−179.37 (19)	C9—C8—C11—O4	−179.53 (16)
C7—C1—C2—O1	−1.7 (3)	O2—Sn1—C12—C13	174.84 (17)
C6—C1—C2—C3	−0.4 (3)	O1—Sn1—C12—C13	−23.33 (19)
C7—C1—C2—C3	177.28 (19)	C16—Sn1—C12—C13	75.8 (2)
O1—C2—C3—C4	−177.65 (19)	N1—Sn1—C12—C13	−106.93 (18)
C1—C2—C3—C4	3.4 (3)	Sn1—C12—C13—C14	170.31 (17)
C2—C3—C4—C5	−2.9 (3)	C12—C13—C14—C15	176.0 (2)
C3—C4—C5—C6	−0.7 (3)	O2—Sn1—C16—C17	−179.92 (16)
C3—C4—C5—Br1	179.46 (16)	O1—Sn1—C16—C17	24.26 (16)
C4—C5—C6—C1	3.6 (3)	C12—Sn1—C16—C17	−77.59 (19)
Br1—C5—C6—C1	−176.54 (16)	N1—Sn1—C16—C17	105.44 (16)
C2—C1—C6—C5	−3.0 (3)	Sn1—C16—C17—C18	62.2 (2)
C7—C1—C6—C5	179.12 (19)	C16—C17—C18—C19	60.7 (3)
C8—N1—C7—C1	179.97 (19)		

### Hydrogen-bond geometry (Å, °)

**Table:**

*D*—H···*A*	*D*—H	H···*A*	*D*···*A*	*D*—H···*A*
O3—H3···O2^i^	0.84	1.77	2.608 (2)	174
O4—H4···O3^ii^	0.84	1.93	2.733 (2)	160

Symmetry codes: (i) −*x*+1, *y*, −*z*+1/2; (ii) −*x*+1, −*y*, −*z*.
